# Consultation rates in cervical screening non-attenders: opportunities to increase screening uptake in GP primary care

**DOI:** 10.1177/0969141315573345

**Published:** 2015-06

**Authors:** Anita Wey Wey Lim, Peter Sasieni

**Affiliations:** Centre for Cancer Prevention, Wolfson Institute of Preventive Medicine, Queen Mary University of London, Charterhouse Square, London EC1M 6BQ

**Keywords:** Cervical screening, primary care, human papillomavirus testing, self-sampling, screening non-attenders, screening coverage

## Abstract

**Objective:**

To estimate the proportion of cervical screening non-attenders presenting to general practice (GP) primary care over one year.

**Setting:**

137 practices in East London, UK.

**Methods:**

Anonymous primary care records were downloaded using EMIS web (clinical software). Cervical screening nonattendance was defined as no recorded smear in the last 3.5 years (women aged 25–49) or 5.5 years (women aged 50–64). The last three consultation entries were used to estimate the proportion of non-attenders who consulted in GP over 3 months and 1 year using the Kaplan-Meier method. Newly registered women were assessed separately. Results were calculated for each practice and the median and interquartile range (IQR) across practices are presented. Heterogeneity was assessed using funnel plots.

**Results:**

Of 261,810 women, 224,313 (86%) had been registered for >1 year. The proportion classified as non-attenders differed between those registered for >1 year (30%, IQR 27%--35%) and within the last year (49%, IQR 40%--57%), suggesting that screening records were less up-to-date in newly registered women. A median of 32% (IQR: 27%--37%) of non-attenders presented over 3 months, and 60% (IQR: 52%--67%) over 1 year. Funnel plots of the proportion of non-attenders presenting by the number of non-attenders showed substantial variation between practices.

**Conclusions:**

Over half of cervical screening non-attenders present to their GP at least once a year, in over 75% of practices. This represents a good opportunity for improving coverage by offering an alternative form of screening, such as self-sampling for human papillomavirus testing.

## Introduction

In England, cervical cancer screening is estimated to save up to 4,500 lives annually.^1^ Nevertheless, 2,482 women were diagnosed^2^ and 742 died from the disease in 2012.^3^ Nonattendance for screening is one of the most important risk factors for developing cervical cancer,^4^ but screening coverage (the proportion of eligible women who are regularly screened) has been falling since the mid 1990s.^5^ Screening uptake is particularly low in young women (aged 25–29),^6^ women who live in deprived areas, and women from ethnic minorities.^7–9^ There is a well-recognized need to develop strategies to increase screening uptake in these women, who are at high risk of developing cervical cancer.

The most commonly cited barriers to cervical screening relate to the pelvic examination (eg. embarrassment, fear of pain) and to practical issues (eg. making appointments, arranging childcare, getting time off work).^10–12^ Self-sampling for high-risk human papillomavirus (HPV) types has the potential to overcome these; women can take their own test in private, at a time and place of their choosing. Several studies^13–19^ have assessed the uptake of self-sampling for HPV testing in screening non-attenders. Approaches have included posting self-sampling kits directly to women^10,13,15,19–23^ writing to women to ask them to order kits,^15–17^ and offering kits door-to-door.^14^ Response rates have ranged between 8.7% and 52.1%, and were highest in women who were offered kits in person (ie. door-to-door). Studies which posted self-sampling kits achieved response rates of around 30% in Northern Europe,^13,16,17,19–23^ but only 6--8% of women returned a self-sample in two UK studies.^10,24^

One approach that has not been assessed is offering self-sampling for HPV testing opportunistically to cervical screening non-attenders in primary care. Women would be offered a self-sampling kit when they present to their GP for any reason. This takes advantage of the fact that the woman is already in clinic (allowing an in person approach), and kits do not need to be ordered or sent (fewer wasted kits and easier for women). The success of this approach depends on a reasonable proportion of screening non-attenders presenting to their GP. Currently little is known about cervical screening non-attenders’ consulting behaviour. The aim of this audit was to estimate the proportion of cervical screening non-attenders that present to their GP and the frequency of attendance over one year, using anonymous electronic primary care records.

## Methods

### Data collection

Anonymous data on women within the cervical screening age range (ie. 25–64) were downloaded from GP computer databases on 31 December 2012 using EMIS Web (Egton Medical Information Systems Ltd, 2010 – one of the main software suppliers to UK GPs). We collected data from all 137 general practices in three primary care trusts (PCTs, now known as CCGs – Clinical Commissioning Groups) in East London (Newham, City and Hackney, and Tower Hamlets) that use EMIS web. Eight further practices were excluded because they used other electronic patient record software. Data included age, date of registration at the practice, details of the last three consultation entries, cervical screening records (dates and codes for the most recent smear entry and for withdrawal or cease from the screening programme), and aggregated ethnicity data for each practice.

### Analysis

In England, women aged 25–49 are invited to cervical screening every 3 years, and women aged 50–64 are invited every 5 years. We therefore classified women’s cervical screening status as follows:

1. ‘Up to date’ – women whose last smear was recorded within 3 years (women aged 25–49) or 5 years (women aged 50–64) prior to download date.
2. ‘Due’ – women whose last smear was recorded >3 and ≤3.5 years (women aged 25–49) or >5 and ≤5.5 years (women aged 50–64) prior to download date.
3. ‘Late’ – women whose last smear was recorded >3.5 and ≤5.5 years (women aged 25–49) or >5.5 and ≤7.5 years (women aged 50–64) prior to download date.
4. ‘Very late’ – women whose last smear was >5.5 years (women aged 25–49) or >7.5 years (women aged 50–64) prior to download date.
5. ‘Never’ – women with no smear recorded and who were not classified as ‘ceased’ (ie. still has cervix, see 6) below)
6. ‘Ceased’ – women who were withdrawn from cervical screening because their cervix had been removed or amputated (eg. hysterectomy) or who had the clinical code “8I6K – cervical smear not indicated” (eg. for women who are terminally ill).
7. ‘Unknown’ – women who had been previously screened but for whom the date was missing from their most recent smear entry.

Women withdrawn from screening by informed choice (ie. had clinical code entries for cervical screening disclaimer forms) were also classified by the above criteria because these women may agree to take a self-sample (eg. if opting out was due to dislike of the pelvic examination).

‘Screening non-attenders’ were defined as women who were more than six months overdue screening (‘late’, ‘very late’) or never screened (‘never’).

When patients move practice there can be delays in transferring their medical records. This could have potentially led to misclassifications in screening status. Therefore, we compared screening status for women who were newly registered within a year of data download with those who had been registered for over one year.

Consultation entries in EMIS include non-consultations such as outpatient letters, laboratory test results, and administration notes. This meant that only limited consultation data were available for women who had non-visit entries for all three. To make best use of the available data we used the Kaplan-Meier method (ie. censoring those who had three non-visit entries at the time of the earliest entry) to estimate the proportion of women who presented over three months and over one year as a function of time, for each practice. Median (and interquartile range [IQR]) of these estimates were then calculated for screening non-attenders (the main group of interest). We also calculated these for all women, and for three other groups of interest:

1. Prompt attenders – screening categories ‘up to date’ and ‘due’
2. Lapsed attenders (ie previously screened and >6 months overdue) – screening categories ‘late’ or ‘very late’
3. Never attenders – screening category ‘never’.

We used funnel plots to explore whether differences between practices in the proportion of screening non-attenders presenting were random or were more likely to be due to other factors (eg. ethnicity, age). To explore heterogeneity in our estimates between practices, we used linear regression to assess whether the proportion of screening non-attenders consulting over 3 months at each practice was affected by the number of women aged 25–64 registered for >1 year, or the proportion of non-attenders registered for >1 year.

We calculated the median and IQR time in years from last cytology test to date of download for screening non-attenders within screening age bands 25–49 and 50–64. All statistical analyses were performed using Stata Statistical Software: Release 12. StataCorp. 2011 (College Station, TX: StataCorp LP). A p value of less than 0.05 was considered statistically significant. All statistical tests were two-sided.

## Results

A total of 261,810 women aged 25–64 were included in the audit. Table 1 shows the practice details for all practices combined and by PCT. Although Tower Hamlets had larger practices, there were fewer women in the cervical screening age range. All three PCTs had ethnically diverse populations; over a third of patients were South Asian at Tower Hamlets and Newham. Overall, 86% (224,313) of women had been registered for at least one year.

**Table 1.** General Practice details by primary care trust.

	Tower Hamlets (*n* = 36)	Newham (*n* = 61)	City and Hackney (*n* = 40)	Total (*n* = 137)
List size				
Median (IQR)	7388 (4395, 10445)	4970 (3191, 8606)	5772 (4440, 9058)	5925 (3944, 9569)
Range	1563--17980	1399--15791	879--13389	879--17980
Number registered >1 year	68,180 (84%)	84,290 (87%)	71,843 (86%)	224,313 (86%)
Ethnicity				
White	38%	26%	47%	–
Black	7%	19%	22%	–
South Asian	37%	42%	7%	–
Other	6%	4%	9%	–
Not recorded	11%	9%	15	
Age in years				
25	6%	4%	4%	11,898 (5%)
26--27	11%	9%	9%	25,401 (10%)
28--37	46%	38%	40%	107,360 (41%)
38--49	22%	28%	28%	67,697 (26%)
50--64	15%	21%	20%	49,454 (19%)
Number of women aged 25--64 years	81,528	96,973	83,309	261,810

IQR=interquartile range.

Table 2 shows how screening status varied according to whether or not women were newly registered within the previous year. More newly registered women were classified as ‘never’ screened, (median 43% [IQR 35%--53%] versus 15% [IQR 13%--19%]), and fewer were ‘up to date’ (median 49% [IQR 41%--56%] versus 63% [IQR 58%--66%]). Of those ever screened, however, a higher proportion of newly registered women (median 89% [IQR 84%--92%]) than other women (median 77% [IQR 74%--80%]) were ‘up to date’ with screening.

**Table 2.** Screening status (median and interquartile range) for women registered ≤1 year and >1 year and for all women.

Screening status	Women registered for ≤1 year Median % (IQR) N = 37,497	Women registered for >1 year Median % (IQR) N = 224,313	All women Median % (IQR) N = 261,810
Up to date	49% (41%--56%)	63% (58%--66%)	60% (56%--65%)
Due	1% (0.3%--2%)	4% (3%--5%)	4% (3%--4%)
Late	3% (2%--4%)	9% (7%--10%)	8% (7%--9%)
Very late	2% (1%--3%)	6% (4%--8%)	6% (4%--7%)
Never	43% (35%--53%)	15% (13%--19%)	19% (16%--23%)
Ceased	1% (0.4%--2%)	3% (2%--4%)	2% (2%--4%)
*Screening non-attenders (late/very late/never)*	*49% (40%--57%)*	*30% (27%--35%)*	*33% (29%--37%)*

IQR = interquartile range.

Note: Additionally, 68 women (0.026%) had unknown screening status.

Table 3 shows the median and IQR of Kaplan-Meier estimates across practices for the proportion of women who consulted over three months and over one year for all women, and according to screening history. A median of 32% (IQR 27%--37%) of screening non-attenders consulted at least once over three months, and 60% (IQR 52%--67%) over one year. In three-quarters of practices at least 37% of screening non-attenders consulted over three months and at least 67% over 12 months. The median proportion of women that consulted was lowest in never attenders (never screened) for both time periods.

**Table 3.** Median (interquartile range) Kaplan-Meier estimates of the proportion of women aged 25--64 consulting over 3 months and 1 year for women registered for >1 year for all women and by screening attendance.

Median (IQR) proportion of women who consulted in the past 3 months	
All women	48% (44%--52%)
By screening attendance	
Prompt attender	56% (51%--60%)
Lapsed attender	37% (29%--43%)
Never attender	28% (22%--33%)
*Screening non-attenders (late/very late/never)*	*32% (27%--37%)*
Median (IQR) proportion of women who consulted in the past 1 year	
All women	77% (73%--82%)
By screening attendance	
Prompt attender	86% (83%--89%)
Lapsed attender	62% (55%--73%)
Never attender	57% (48%--64%)
*Screening non-attenders (late/very late/never)*	*60% (52%--67%)*

IQR=interquartile range.

Prompt attender=screening categories ‘up to date’ and ‘due’.

Lapsed attender= screening categories ‘late’ and ‘very late’.

Never attender= screening category ‘never’.

**Figure 1.**
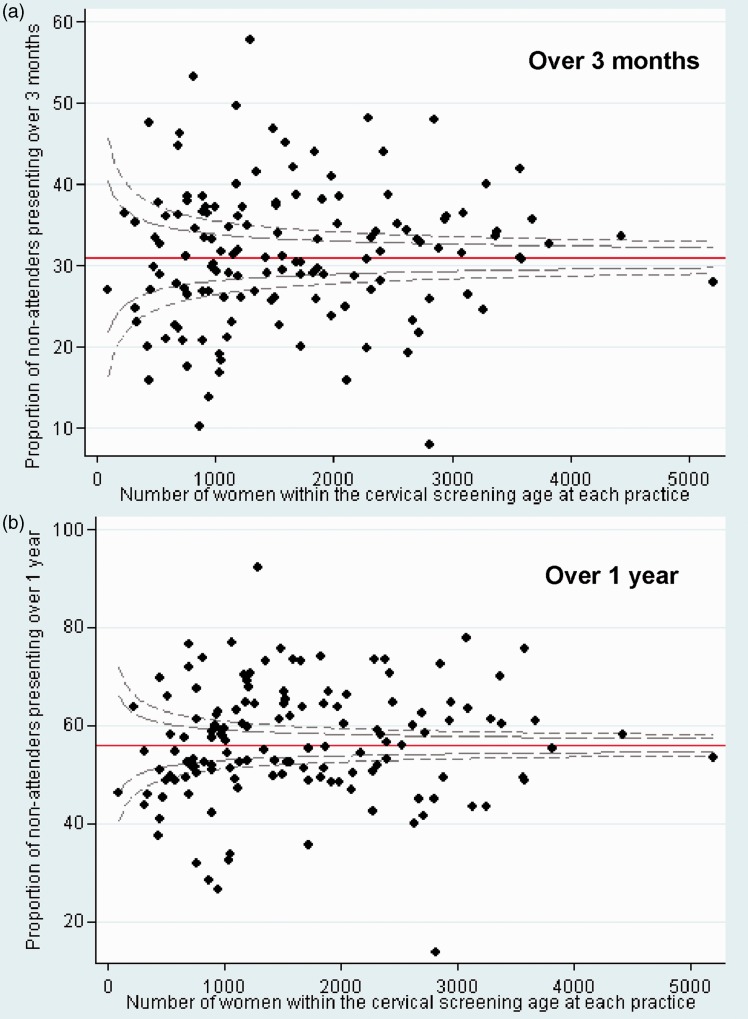
Funnel plots showing the proportion of screening non-attenders presenting by the number within the cervical screening age at each practice (for women registered >1 year). Control limits indicated by the dashed lines are set at 95% and 99.8%.

Only 9% (5991/68,715) of screening non-attenders were censored because there were insufficient data to determine if they had consulted in the past 12 months (ie. all three consultation entries within 12 months were non-visit entries). For screening non-attenders aged 25–49, the median time since last smear was five years (IQR 4–7) and for those aged 50–64 this was nine years (IQR 7–13). Figure 1a and b both show a roughly symmetrical funnel shaped distribution for the proportion of screening non-attenders that consulted over each time period by the number of women in the cervical screening age at each practice. However, approximately two thirds were scattered outside of the 99.8% control limits (dotted lines) for both periods, indicating substantial heterogeneity between the practices and hence the need to report median and IQR in Tables 2 and 3.

We also looked at practice-specific factors that might predict the proportion of screening non-attenders presenting over three months. The proportion of screening non-attenders presenting was unaffected by the number of women aged 25–64 at each practice registered >1 year (p=0.448) and by the proportion of screening non-attenders registered >1 year (p=0.238).

## Discussion

### Main findings

Over half of cervical screening non-attenders in East London present to their GP at least once a year, and approximately a third over three months. This suggests that a reasonable proportion of screening non-attenders could be offered opportunistic self-sampling for HPV testing when they present to their GP. The proportion varied substantially between practices.

Cervical screening data in GP medical records appears to be unreliable within a year of registration, as the proportion of women who were classified as ‘never’ screened was much higher in comparison with women registered for more than a year. The fact that a higher proportion of newly registered women who had been screened previously were ‘up to date’ could indicate that registration visits are being used as an opportunity to carry out or remind women about cervical screening. Cervical screening non-attenders consult their GP less often (60% within one year) than do screening attenders (86% within one year), but it is not the case that non-attenders do not engage with the National Health Service.

### Strengths and weaknesses

A key strength of this study is the large and unbiased database. Excluding women who were newly registered in the last year in the main analysis allowed us to calculate more accurate estimates within screening categories. Because we only had partial consultation data, the proportion of women who presented is likely to have been under- or over-estimated. However, the use of Kaplan-Meier ensured that all available information was used for our estimates. Only 9% of screening non-attenders were censored (because they had non-consultation entries for all available entries), suggesting that any under- or over-estimation was small. Because our audit was limited to practices in a single urban area of London with a high proportion of South Asians, our data may not be representative of the rest of England. It is known that a high proportion of South Asians are cervical screening non-attenders,^8^ but we were not able to study consulting behaviour separately in South Asians and White screening non-attenders.

### Comparison with other studies

We are not aware of any studies that have specifically examined GP primary care consultation rates in cervical screening non-attenders. However, a questionnaire study in Denmark found that GP consultation rates were lower in non-attenders who had never been screened than those who had attended screening at least once (64% of never attenders consulted over one year compared with 83% of ever attenders, p < 0.0001).^25^

### Implications

A substantial proportion of cervical screening non-attenders could potentially be offered self-sampling for HPV testing when they present to their GP. This has the potential to increase screening coverage. Studies have shown that a high proportion (often >80%) of women who test HPV positive on a self-sample attend for follow up investigations (eg. cytology or colposcopy).^23,26,27^ For opportunistic self-sampling to work, not only do screening non-attenders need to attend their GP, but the health professional consulted needs to invite the women to take a self-sample. This may not always be possible due to time constraints (the average GP consultation lasts around 12 minutes^28^) or illness.

Based on these results we are further investigating the potential for opportunistic offering of self-sampling for HPV testing in pilot study (to assess feasibility and acceptability). This will be followed by a large randomized controlled trial.

## Conclusion

Over one year, over half of cervical screening non-attenders could be offered self-sampling for HPV testing opportunistically when they present to their GP. This approach should be explored further as a means to increase cervical screening coverage.

## Supplementary materials

A dataset is available from the corresponding author on request.
